# Supplementary figures and images for: Candida albicans translocation through the intestinal epithelial barrier is promoted by fungal zinc acquisition and limited by NFκB-mediated barrier protection
Source: PLoS Pathog. 2024 Mar 1;20(3):e1012031. doi: 10.1371/journal.ppat.1012031 (PMC10907035; doi:10.1371/journal.ppat.1012031)

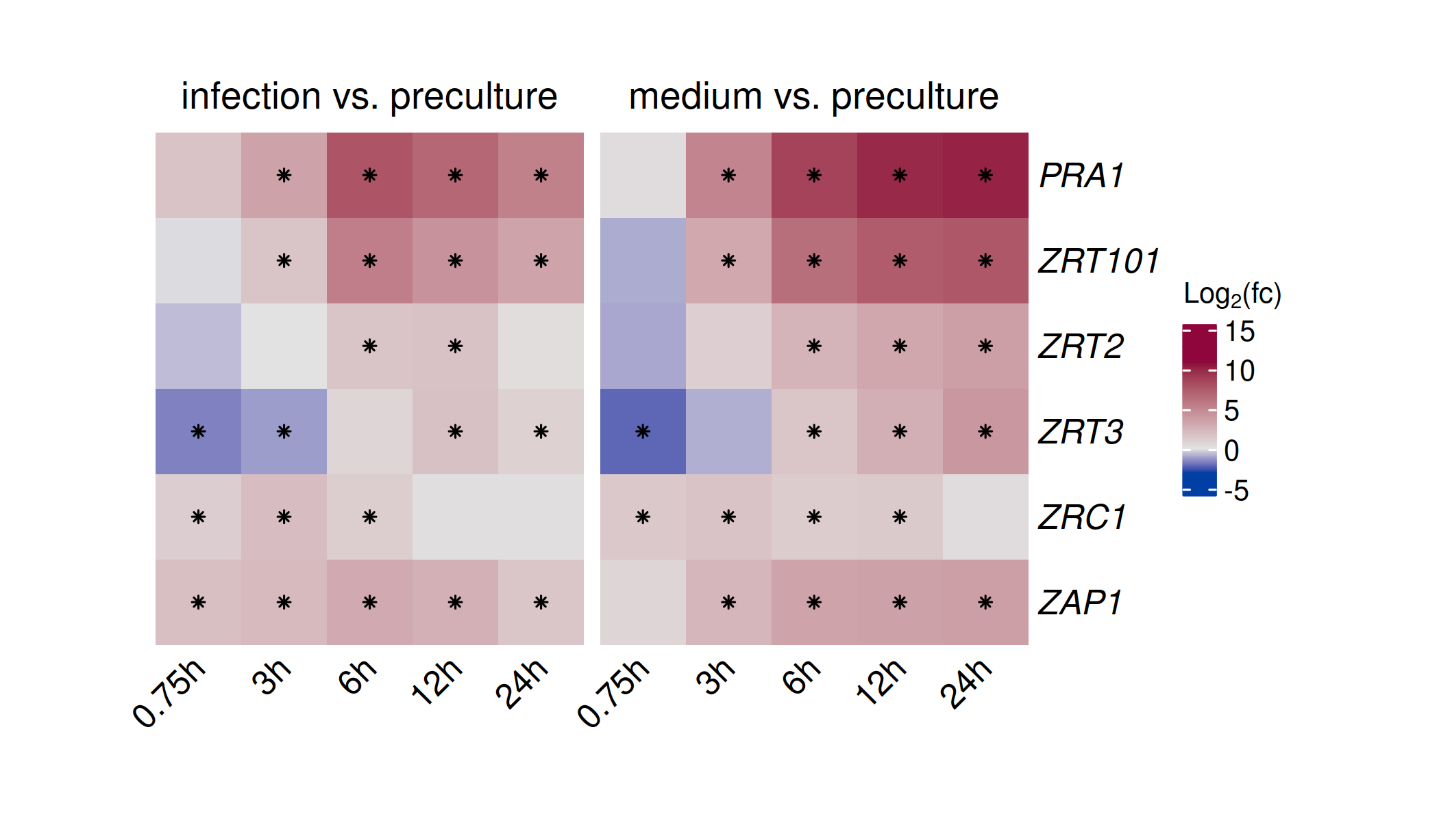

Supplement: S1 Fig — Expression of the genes involved in zinc transport (ZRT101, ZRT2, ZRT3, ZRC1), zinc scavenging (PRA1), and regulation of zinc acquisition genes (ZAP1). Log2(fold-change) compares infected samples to the yeast pre-culture conditions on the left and medium-only samples to the yeast pre-culture conditions on the right. Asterisks indicate time points with significantly expression changes (DESeq2 p < 0.05). (TIF) [file ppat.1012031.s001.tif]

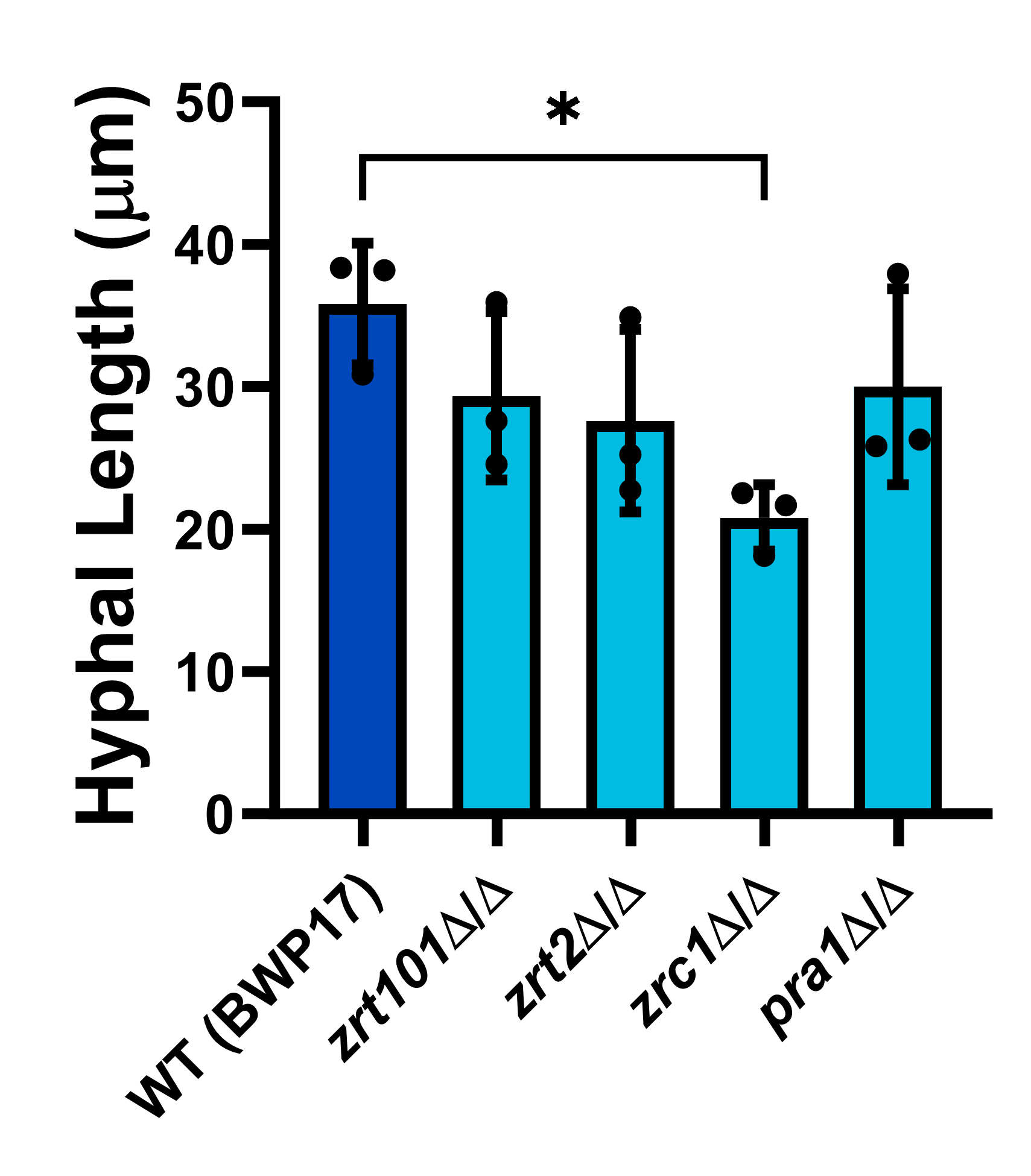

Supplement: S2 Fig — Loss of ZRT101, ZRT2, or PRA1 did not significantly impact hypha formation. However, loss of ZRC1 significantly decreased hyphal length in C. albicans in cell culture medium after 6 h. All values are shown as the mean with standard deviation. Data were compared using a one-way ANOVA with a post-hoc Dunnett’s multiple comparisons test. Statistical significance: *, P ≤ 0.05. (TIF) [file ppat.1012031.s002.tif]

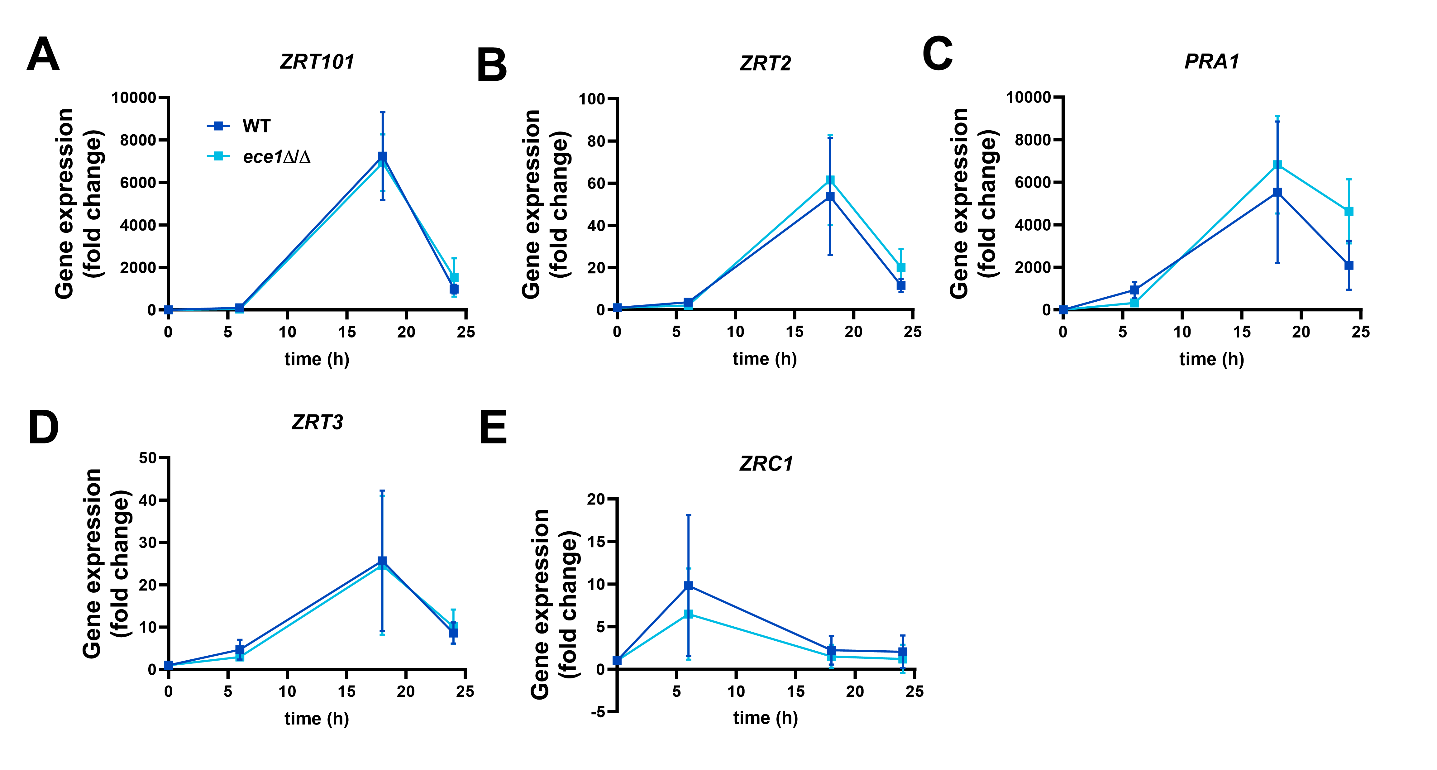

Supplement: S3 Fig — Fold change in gene expression in C. albicans WT (BWP17) and ece1Δ/Δ strains during incubation in cell culture medium for (A) ZRT101, (B) ZRT2, (C) PRA1, (D) ZRT3, and (E) ZRC1. All values are shown as the mean with standard deviation. (TIF) [file ppat.1012031.s003.tif]

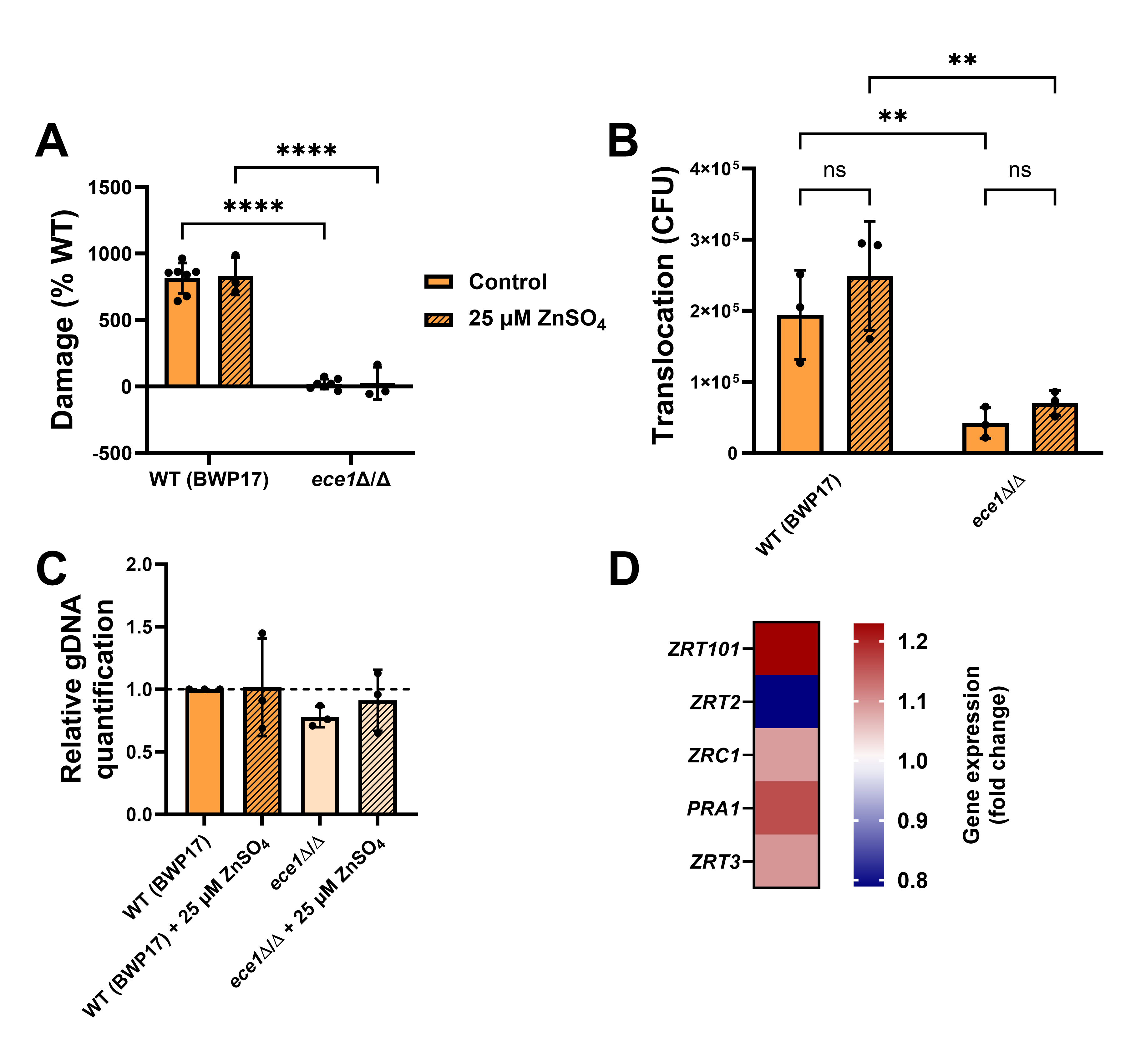

Supplement: S4 Fig — (A) Host-cell damage in the absence or presence of 25 μM exogenous ZnSO4 for the WT mutant (same data presented in Fig 2D) and the ece1Δ/Δ strain. (B) Fungal translocation of the WT(BWP17) and ece1Δ/Δ strains with or without the addition of 25 μM ZnSO4. (C) Relative quantification of fungal gDNA during infection of IECs. All samples are compared to the WT(BWP17) without added zinc. (D) Fold change in normalized gene expression of the ece1Δ/Δ strain compared to WT(BWP17) at 24 h during infection of IECs with addition of 25 μM ZnSO4. Gene expression was normalized to ACT1 as a housekeeping gene. All values are shown as the mean with standard deviation. (TIF) [file ppat.1012031.s004.tif]

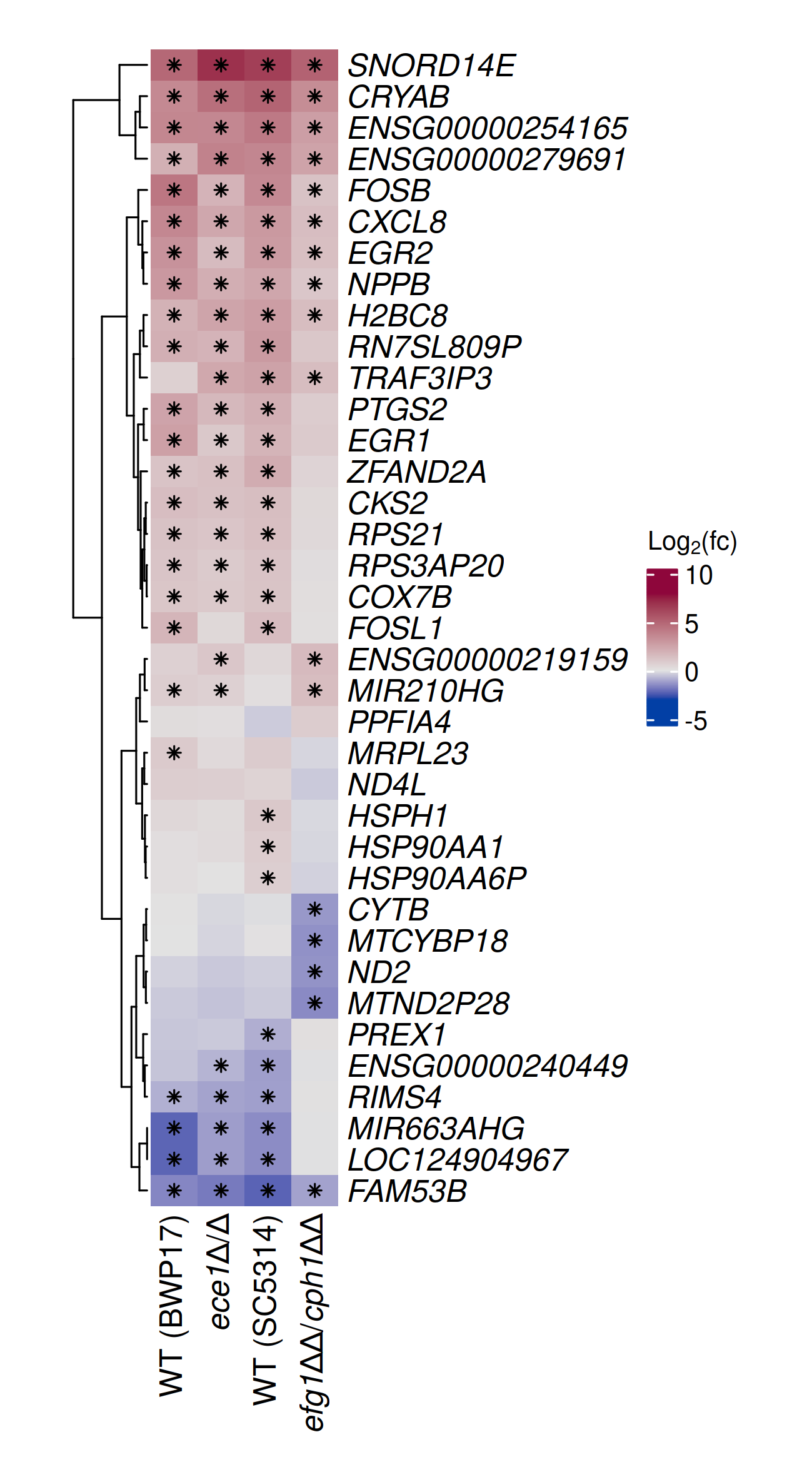

Supplement: S5 Fig — Genes differentially expressed when comparing the non-damaging ece1Δ/Δ and the non-filamentous efg1ΔΔ/cph1ΔΔ strains to their respective WT strains (ece1Δ/Δ compared to WT (BWP17) and efg1ΔΔ/cph1ΔΔ compared to WT (SC5314)). The data are shown as the Log2(fold-change) of infected cells with the different strains compared to uninfected IECs. Asterisks indicate genes with statistically significant differences in expression (DESeq2 p < 0.05). (TIFF) [file ppat.1012031.s005.tiff]

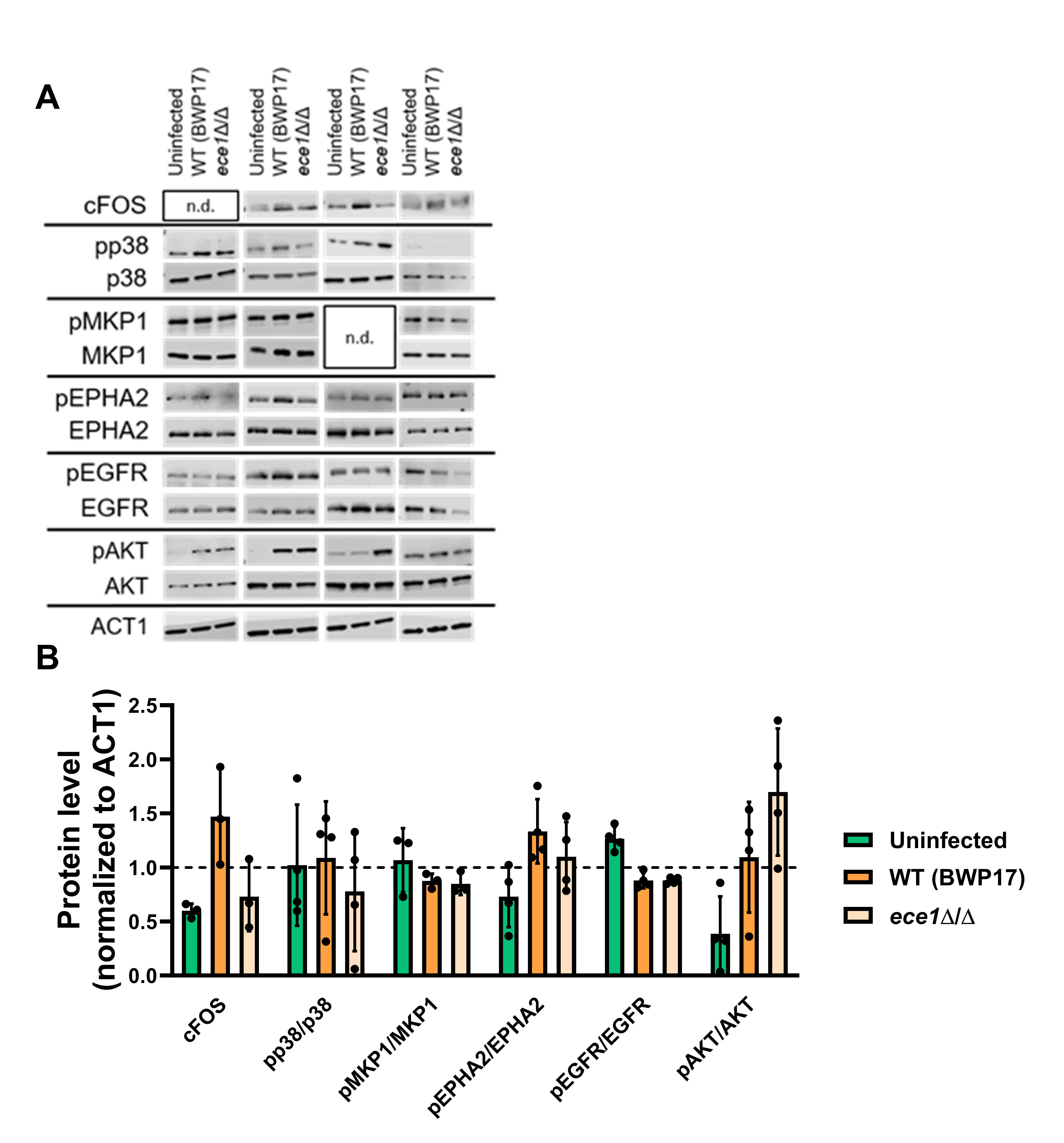

Supplement: S6 Fig — (A) Confluent, differentiated C2BBe1 cells were infected with WT (BWP17) and ece1Δ/Δ C. albicans for 6 h and the protein content was sampled. Proteins involved in the damage response of oral epithelial cells were detected with ACT1 serving as a control. n.d. = not determined. (B) Protein levels normalized to actin. For p38, MKP1, EPHA2, EGFR, and AKT the normalized protein level for the phosphorylated protein is presented relative to the total respective protein level. (TIF) [file ppat.1012031.s006.tif]

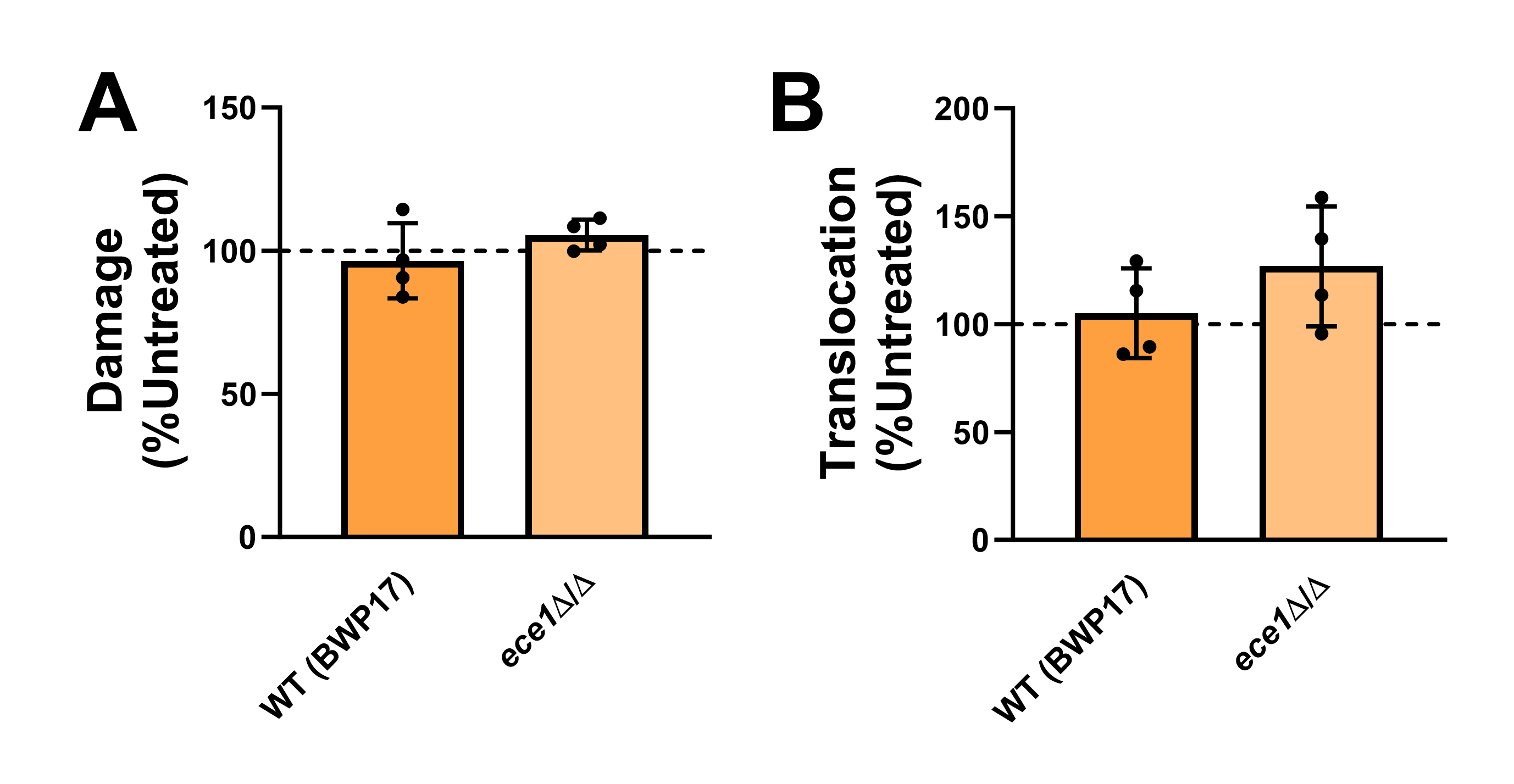

Supplement: S7 Fig — C2BBe1 cells were treated with a DMSO vehicle control and infected with the WT or ece1Δ/Δ C. albicans strains. There were no significant changes in (A) host cell damage or (B) fungal translocation. All values are shown as the mean with standard deviation. (TIF) [file ppat.1012031.s007.tif]

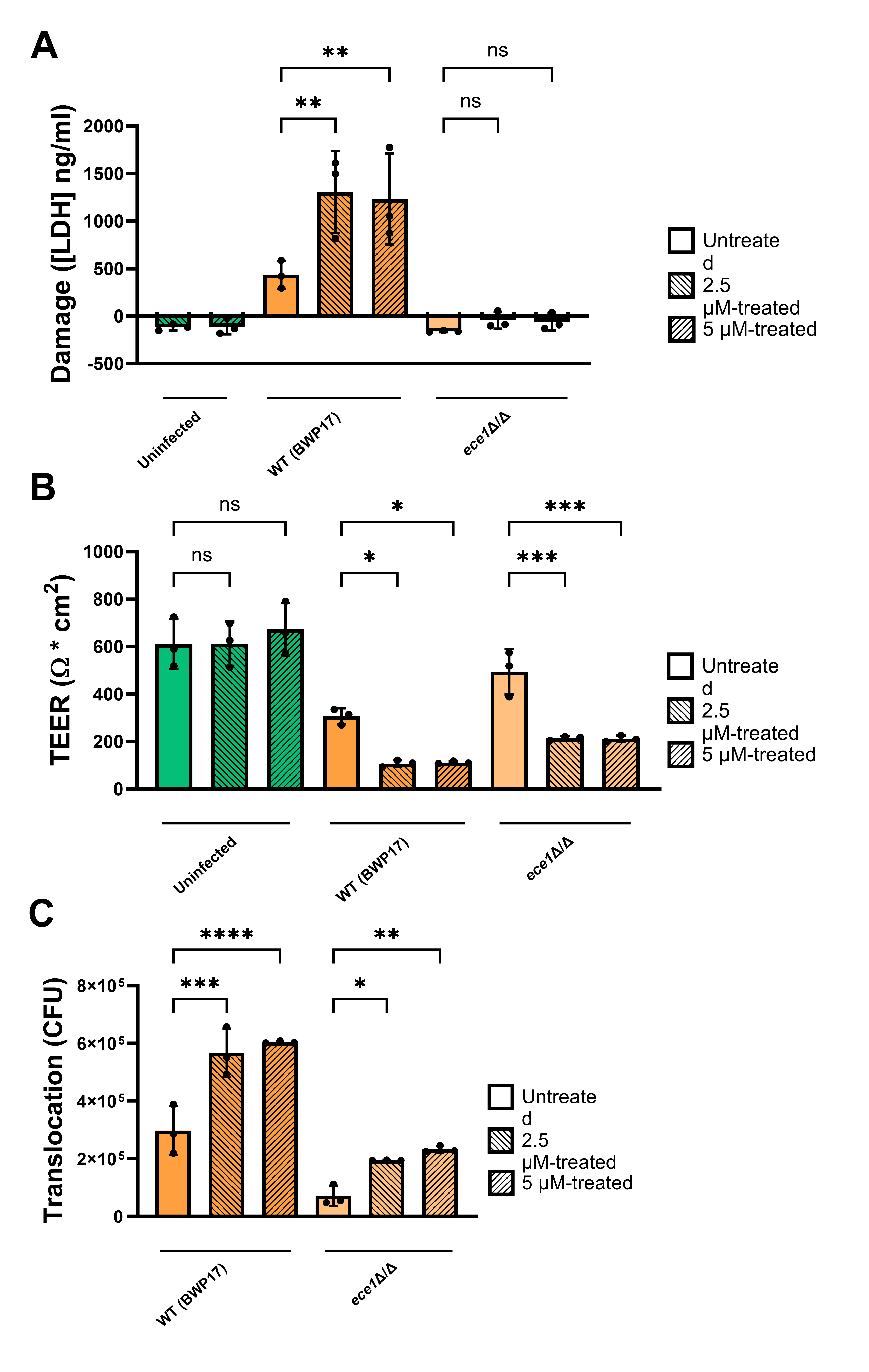

Supplement: S8 Fig — (A) Inhibition of NFκB activation using the NFκB inhibitor SC75741 at concentrations of either 2.5 or 5 μM increased the damage of WT (BWP17) C. albicans, but not for the ece1Δ/Δ strains. LDH release was adjusted by subtracting the release from uninfected and untreated host cells. (B) NFκB inhibition using either concentration also decreased the barrier integrity during infection with both WT and ece1Δ/Δ strains. (C) Fungal translocation was significantly increased during infection with both WT and ece1Δ/Δ C. albicans upon inhibition of NFκB using both concentrations of SC75741. These results match those obtained with the high-affinity NFκB inhibitor quinazoline. All values are shown as the mean with standard deviation. Host-cell damage (A), barrier integrity (B), and fungal translocation (C) data were compared using a one-way ANOVA with a post-hoc Šidák’s multiple comparisons test. Statistical significance: *, P ≤ 0.05; **, P ≤ 0.01; ***, P ≤ 0.001; ****, P ≤ 0.0001. (TIF) [file ppat.1012031.s008.tif]

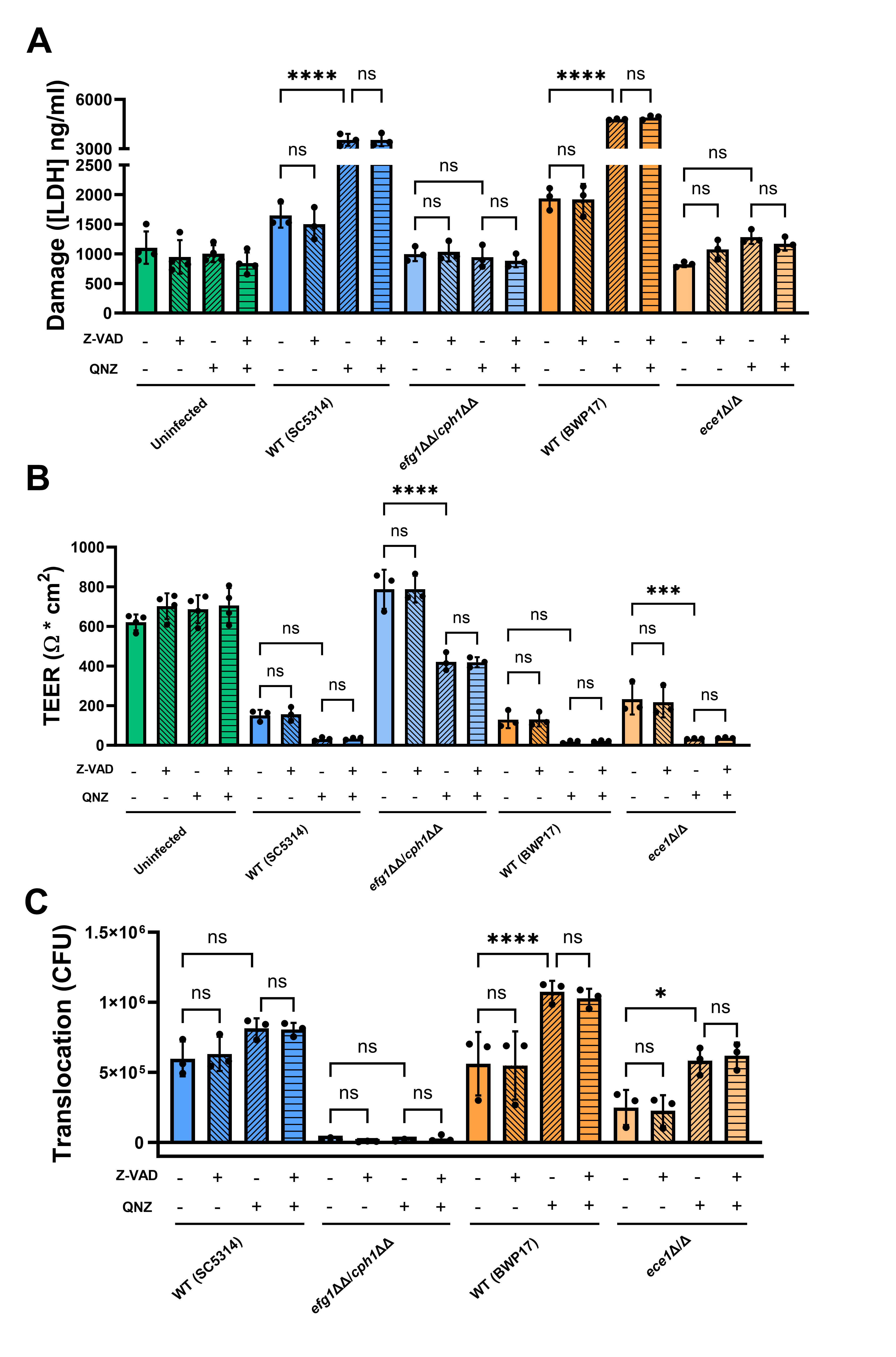

Supplement: S9 Fig — Treatment with a pan-caspase inhibitor (Z-VAD) had no significant effect on (A) host-cell damage, (B) barrier integrity, or (C) fungal translocation when used alone or in combination with the NFκB inhibitor quinazoline (QNZ). All values are shown as the mean with standard deviation. Host-cell damage (A), barrier integrity (B), and fungal translocation (C) data were compared using a one-way ANOVA with a post-hoc Šidák’s multiple comparisons test. Statistical significance: *, P ≤ 0.05; ***, P ≤ 0.001; ****, P ≤ 0.0001. (TIF) [file ppat.1012031.s009.tif]

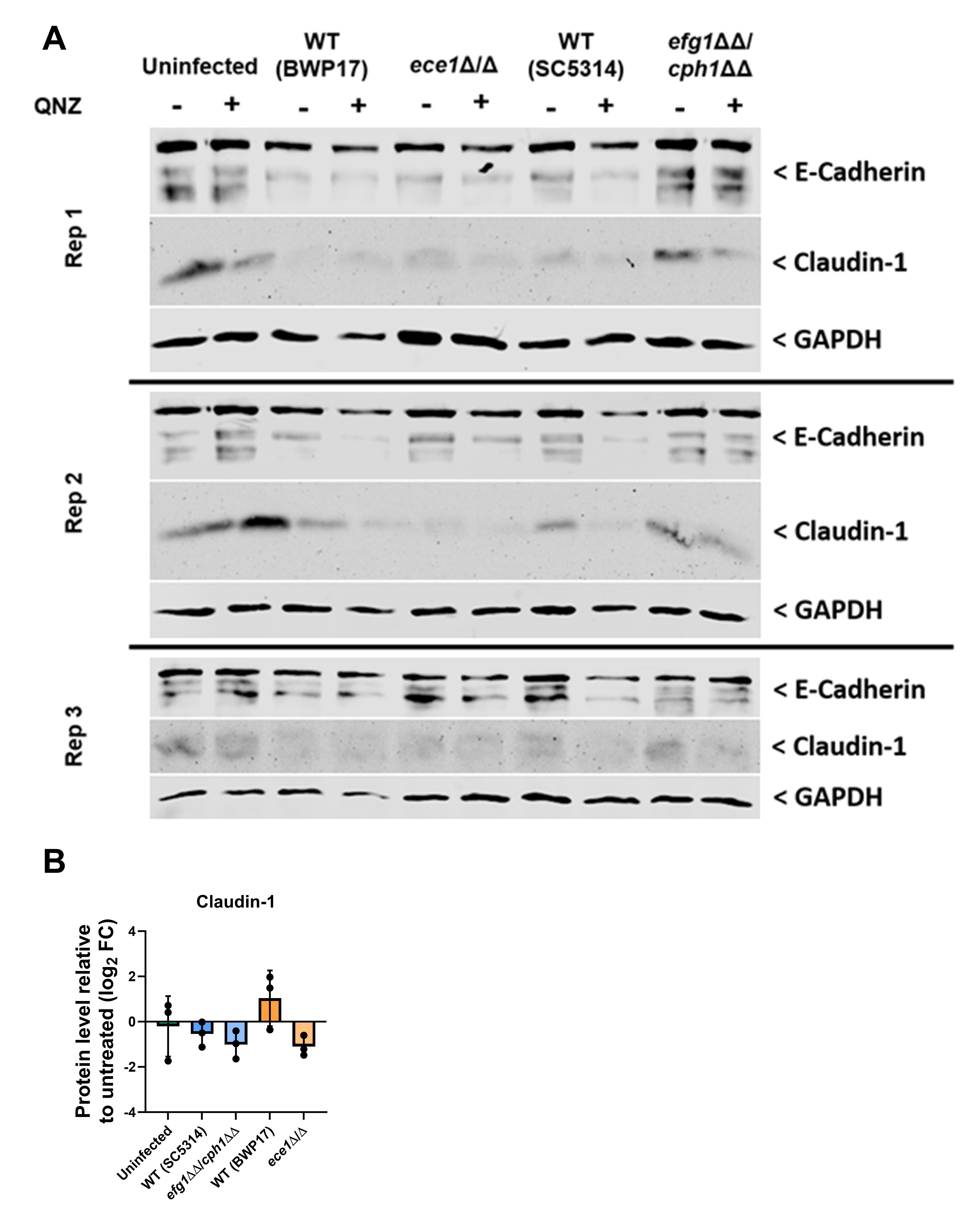

Supplement: S10 Fig — Confluent, differentiated C2BBe1 cells were infected with WT ece1Δ/Δ, and efg1ΔΔ/cph1ΔΔ C. albicans for 24 h. Samples were either untreated or treated with an NFκB activation inhibitor (QNZ) and the protein content was sampled. (A) Proteins that make up tight and adherens junctions (E-cadherin and claudin-1) were detected with GAPDH serving as a control. (B) Claudin-1 protein levels normalized to GAPDH and presented relative to levels in untreated C2BBe1 cells. QNZ treatment further increased degradation of claudin-1, even during infection with efg1ΔΔ/cph1ΔΔ and ece1Δ/Δ. All values are shown as the mean with standard deviation. (TIF) [file ppat.1012031.s010.tif]
