# Supplementary material for: Candida albicans translocation through the intestinal epithelial barrier is promoted by fungal zinc acquisition and limited by NFκB-mediated barrier protection
Source: PLoS Pathog. 2024 Mar 1;20(3):e1012031. doi: 10.1371/journal.ppat.1012031 (PMC10907035; doi:10.1371/journal.ppat.1012031)
Supplement: Attachment — Submitted filename: Response Letter.pdf [file ppat.1012031.s014.pdf]

Dear Editors,

We would like to thank you all and the reviewers for the detailed and constructive comments.

Our manuscript had been reviewed by three referees who found our work to be of interest, but also raised some concerns. To address these issues, we have performed additional experiments and analyses as requested.

Furthermore, we have included in-text changes to correct any mistakes pointed out by the reviewers and expand the discussion to better contextualize and interpret our data.

We have provided point-by-point responses to each comment from all three reviewers on the following pages and believe that we were able to deal with all critical points sufficiently.

We hope that these revisions are satisfactory and allow our manuscript to be considered for publication.

We look forward to your response.

Best regards,

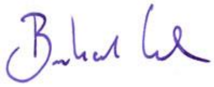

Bernhard Hube

Our replies (black) to the reviewers' comments (blue) are below:

***Line numbers refer to the manuscript copy with track changes.**

Comments by Reviewer 1:

This manuscript by Sprague et al employed the dual RNAseq strategy to explore the pathogen and host factors during intestinal epithelial cell infection by C. albicans. The authors plotted the differentially expressed genes in both fungal and host cell compartments and found zinc acquisition and NFκB pathway as key processes, respectively. Although dual RNAseq is a powerful tool in studying host-pathogen interactions, the experimental design has deviated from the scenario of Candida intestinal translocation, which hampers the data value.

We appreciate the feedback from **Reviewer 1** and are aware of the technical limitations of our model system. We acknowledge these limitations in the manuscript and have added further contextualization of our results and how they relate to the *in vivo*/clinical situation into the Discussion (Lines: 518-525; 589-595).

In the dual RNAseq experiment, before the fungal cells and C2BBe1 cells seeded into the DMEM medium, fungal cells were cultured in the YPD broth. With the transition from the rich medium (YPD) to the nutrient-limit medium (DMEM), the fungal cells would experience a drastic metabolic rewiring, which may affect the sensitivity of RNAseq in detecting differentially expressed genes to host cell. In contrast, host cells were consistently maintained in DMEM environment and the only difference at the start of the experiment was the depletion of serum. This might explain why transcription profiles of fungal compartment were generally similar with or without host cells at each time points. It is challenging to prepare and quantify C. albicans cells in DMEM medium for this experiment since a lot of the cells would develop to hyphae and become clumpy. But the authors should discuss this when explaining the pattern of fungal DEGs and need to be aware of this limitation before claiming "fungal response was largely independent of the presence of host cells" (line108).

We agree with **Reviewer 1** that nutrient-rich YPD precultures for *C. albicans* cells prior to seeding them in DMEM with or without host cells likely affects the sensitivity of identifying DEGs specific for the presence of host cells. This adaptive response to the new, nutrient poor medium from a nutrient rich pre-culture was also described previously in Fradin *et al*., 2003 (*Mol Microbiol*) (DOI: 10.1046/j.1365-2958.2003.03396.x.). This study showed that the pre-culture condition (YPD vs. Lee's medium) had an effect on gene expression upon incubation in human blood after 10 min, but this effect was already decreased after 30 min of incubation. Additionally, the study found that many genes had similar expression patterns upon incubation in human blood, independent of the pre-culture conditions. We have chosen our earliest time point for the RNA sequencing dataset to be 45 min, to limit such effects of the preculture conditions of *C. albicans* on the transcriptional response. Taken together, transcriptional changes in *C. albicans* due to the medium switch might lead to similar profiles for the control and infection samples, but these would be fairly rapid and do not explain similar profiles at later time points.

The similarity of fungal transcription profiles with or without host cells are likely rather due to the presence of strong hypha-inducing conditions in both samples (37°C, alkaline pH, CO_2), which induces a filamentation-specific transcriptional program in both samples. Such expression patterns can be seen, for example, in the core filamentation genes identified in Martin *et al*., 2013 (*PLOS One*) (DOI: 10.1371/journal.pone.0058613). Shown below is a heatmap depicting the $\log_2$ fold change in expression in *C. albicans* samples (medium only on the left, IEC infection on the

right) compared to the YPD pre-culture for the 4 most highly upregulated genes in the core filamentation response. There is a strong upregulation of all 4 genes across all time points, regardless of the presence of host cells.

	**Medium (time point x) vs. YPD (0 h)**					**Infection (time point x) vs. YPD (0 h)**				
	**0.75 h**	**3 h**	**6 h**	**12 h**	**24 h**	**0.75 h**	**3 h**	**6 h**	**12 h**	**24 h**
ALS3	5.61	5.28	5.16	6.82	3.79	6.79	6.76	5.45	6.75	4.27
ECE1	11.12	11.71	10.97	13.78	12.20	12.14	13.09	11.53	13.80	12.44
HWP1	10.57	11.86	12.01	13.50	12.11	11.47	11.67	11.18	12.29	11.15
IHD1	6.61	7.93	7.06	8.10	9.30	7.63	7.26	6.88	8.14	8.83

log_2 fold change

-15 0 15

The presence of strong triggers for filamentation in both the infected and medium-only control samples is already mentioned in the Discussion (Lines: 461-463). We have expanded the Discussion to address the switch from YPD to DMEM and included this information for the interpretation of our results (Lines: 463-467). In addition, we have added the phrase "in this experimental model" to our statement in Line 110.

The authors used C2BBe1 cell line as a surrogate for intestinal barrier and found genes in zinc acquisition pathway differentially expressed during infection. However, such in vitro scenario might not be that relevant to the real world. In the in vitro system, the DMEM media is zinc-free, fungal genes related to zinc acquisition are highly upregulated and very sensitive to the zinc from those lysed host cells. In contrast, the human intestinal lumen is normally a zinc-rich environment, this is different from other infectious sites such as the oral cavity. In the gut, C. albicans does not need to damage epithelial cells to acquire zinc. Will these fungal genes be differentially expressed during infection in the presence of zinc? A qPCR test would help answer this question.

We have performed the requested experiment. A heat map comparing the expression of the zinc-related genes in the *ece1*Δ/Δ strain to the WT (BWP17) after 24 h of co-incubation with IECs and 25 µM $ZnSO_4$ has been added to SI Fig 3F. The data show that though the expression is more similar between the strains when added zinc is not present, the *ece1*Δ/Δ strain shows a trend towards higher expression. Likely this is due to a shift in the zinc starvation response that would be more apparent at even later time points which are not achievable in our model system.

We agree that *in vitro* systems can never entirely reflect the *in vivo* scenario. However, we still believe that our data are relevant in a clinical setting as during the onset of systemic infection, *C. albicans* is no longer in contact with the zinc present in the intestinal lumen and would rather need to acquire zinc from host cells and normally sterile environments of the host during epithelial invasion and translocation. This is referenced in the Discussion and we have included reference to the new qPCR data as well (Lines: 516-518).

The loss of function of NFκB was only achieved via chemical inhibitor. The authors should address the specificity level of QNZ on inhibiting NFκB, and use another approach (another inhibitor, knock

down the gene, etc.) to perform one key experiment (damage or translocation) and show a consistent result.

We apologize if the data were not clearly mentioned or stressed enough in the main text, but experiments using another NFκB inhibitor (SC75741) with a different mode of action from QNZ were included as SI Figure 7. We performed host-cell cytotoxicity, barrier integrity, and fungal translocation experiments with both the WT (BWP17+CIp30) and the *ece1*Δ/Δ strain with two concentrations of SC75741. We see a consistent result for all phenotypes tested with even stronger evidence for a damage-independent mechanism of NFκB activation in limiting fungal translocation as the *ece1*Δ/Δ showed no change in host-cell damage despite increased translocation (SI Figure 7A, C). We have changed the text in order to make it clearer that the data in SI Figure 7 were performed with a second NFκB inhibitor (Lines: 363-366).

To define filamentation-specific and damage-specific response host genes, it is better to have the fungal mutations, ece1Δ/Δ and efg1ΔΔ/cph1ΔΔ, in the same strain background. Although BWP17 is derived from SC5314, there are still genetic differences between the two isolates. The authors can briefly address this when discussing host response genes.

We have added text to the Results to address this (Lines: 288-291).

The legend of Figure 1B, “Gene ratio represents the proportion of genes within each category that were significantly differentially expressed during infection compared to IECs only.” This figure is about the DEGs in Candida, should be “compared to C. albicans only”.

We apologize for the mistake; the legend has been changed accordingly.

Figure 2D, show individual data points as figure 2A-C and other figure panels, not just the error bar.

Individual data points are now included in Figure 2D.

Figure 2E, image quality needs to be improved. May add arrows or dash line to point out the key information or quantify the hyphal length or growth via imaging software.

We have performed new experiments to generate improved microscopic images. New images have been added to Figure 2E with additional calcofluor white staining to make the *C. albicans* cells more clearly visible, especially for the *zrt101*ΔΔ/*zrt2*ΔΔ strain without added zinc. The figure caption has also been changed to reflect the new images.

Comments by Reviewer 2:

This study by Sprague et al analyzes the fungal and host pathways involved in infection, invasion, damage, and translocation of C. albicans through cultured human intestinal epithelial cells (C2BBe1). Several approaches were used to interrogate this interaction; first, a dual RNA-sequencing approach was used to profile both fungal and epithelial transcriptional changes throughout the infection. For C. albicans, gene expression was compared between fungi cultured in media alone vs infection of IECs. This analysis revealed a number of transcriptional changes specifically associated with IEC infection, which were mainly differences in transcripts associated with nutrient acquisition. The study focuses specifically on alterations in the zinc acquisition and detoxification pathways for functional analysis. Genes encoding zinc acquisition transporters and zincophores were significantly decreased in expression during IEC infection compared to media

only conditions, suggesting that C. albicans are more zinc limited in media alone compared to with IECs. Disruption of genes encoding zinc transporters, ZRT101 and ZRT2, together lead to a significant decrease in cytotoxicity, which was rescued by the addition of exogenous zinc. This decrease in cytotoxicity was likely due to a significant growth defect of the zrt101Δ/Δ zrt2Δ/Δ during infection, which was rescued by addition of zinc. The authors test whether the cytolytic toxin, Candidalysin, alleviates the zinc starvation response, likely through the release of IEC zinc. This analysis shows that specifically during co-culture with IECs does the ece1Δ/Δ strain show a significant increase in expression of zinc starvation-associated genes. The authors then investigate the transcriptional response of the IECs to infection, finding changes that are largely similar to what has been observed in other C. albicans/epithelial interactions studies (though with some distinctions that were not explored in detail here). The study then focuses specifically on the role of NFkB activation in C. albicans damage and translocation. Transcripts associated with NFkB signaling are significantly upregulated at 6+ hours post infection, which coincides with C. albicans invasion and damage of IECs. Additional transcriptional profiling experiments with hyphal deficient and ece1Δ/Δ mutants show that NFkB transcripts were activated regardless of hyphal and Candidalysin production. The authors use a NFkB activation inhibitor to functionally interrogate whether NFkB signaling was differentially required for blocking all C. albicans strains from damaging and translocating through the IEC layer. They find that NFkB inhibition exacerbates WT C. albicans cytotoxicity and translocation, but has differential effects on the hyphal deficient and ece1Δ/Δ mutants. NFkB inhibition promotes ece1Δ/Δ but not the hyphal deficient mutant, translocation. The authors then show that NFkB inhibition leads to loss of cell surface e-cadherin localization in cells coincubated with the ece1Δ/Δ.

Overall, this is a comprehensive study that reveals important mechanisms behind C. albicans infection and translocation through intestinal epithelial cells. These results are significant given that intestine is an important reservoir for C. albicans, and that transit through the intestinal epithelium is thought to be a main route of invasion that seeds disseminated infections. Efforts to interrogate the role of both C. albicans and IEC pathways are impressive and add to the rigor of the study. I believe this is a useful study for the field of C. albicans/epithelial interactions.

We thank **Reviewer 2** for the positive assessment of our study and agree that our data will be useful for the field of *C. albicans* interactions with epithelial cells.

The role for zinc acquisition during IEC infection is interesting. The ece1Δ/Δ mutant has increased expression of zinc transporters during infection. Is this a biologically meaningful result? That is, does the ece1Δ/Δ mutant have a growth defect compared to WT that is better rescued by the addition of zinc?

In general, the *ece1*Δ/Δ strain does not have any growth or morphology defects compared to the WT on IECs in the initial stages of infection. Invasion of IECs and invasive hyphal length are similar between the strains (Allert *et al*., 2018 (*mBio*); DOI: 10.1128/mBio.00915-18). However, these measurements are taken only after 6 h of incubation, before we see a difference in the transcriptional zinc starvation response. A more complex model system that allows for monitoring *C. albicans* invasion of IECs at even later time points than we have in the current study would likely be needed to determine whether this increased starvation response is associated with a growth defect.

We have adjusted our phrasing in Lines 34, 35, 92, and 494-495 to specify that our data point to a connection between fungal *ECE1*-mediated host-cell damage and the acquisition of host zinc.

Further contextualization for the results with zinc acquisition by *C. albicans* during IEC infection has also been added to the Discussion (Lines: 518-525).

The discussion around the QZN-treatment experiments are overinterpreted or overstated. The text repeatedly states that QZN has little to no impact on ece1Δ/Δ cytotoxicity, but there appears to be an increase in LDH during QZN treatment, which may be biologically significant given the severe defect in ece1Δ/Δ without QZN (basically zero cytotoxicity in 2D). I do not agree that the impact of QZN on ece1Δ/Δ translocation is completely due to a cytoxicity-independent pathway. If NFkB inhibition promoted fungal translocation in a damage -independent manner, wouldn't you expect to see increased translocation of the hyphal deficient strain? Why is there no E-cadherin mis-localization with the hyphal-deficient strain?

We agree that the cytotoxicity data with the QNZ inhibitor do show a slight (though not significant) increase in host-cell damage by the *ece1*Δ/Δ strain (Figure 5A), but this is not present in the data using the SC75741 NFκB inhibitor (SI Fig 7A). The SC75741 inhibitor results in increased translocation of the *ece1*Δ/Δ strain with no corresponding change in host-cell damage. We have changed the text to refer to the QNZ effect on *ece1*Δ/Δ cytotoxicity being not statistically significant (Lines: 349-351) and to better stress the data with the second NFκB inhibitor (Lines: 363-366).

Regarding the non-filamentous *efg1*ΔΔ/*cph1*ΔΔ strain, we do not expect any translocation even with NFκB inhibition because the process as a whole still requires hypha formation (Allert *et al*., 2018 (*mBio*); DOI: 10.1128/mBio.00915-18). We refer in the manuscript text to the requirement of hypha formation (Line: 358-360). To further clarify, we have added a reference for *efg1*ΔΔ/*cph1*ΔΔ being non-filamentous in the respective paragraph (Line: 358-360) and have rephrased the sentence in Line 370.

Use of QNZ itself does not decrease the integrity of the intestinal barrier (Fig 5C) and the non-filamentous strain does not invade the cells or produce any hypha-associated factors. We hypothesize that activation of NFκB may increase the IECs' ability to protect from degradation of the cell-cell junction proteins, but that hypha formation and the associated genes expressed during this process are still necessary to degrade the barrier enough to allow for fungal translocation (Lines: 415-419).

The change in E-cadherin protein levels are relatively unchanged according to Fig 5D, but are discussed as if there are meaningful differences between the *C. albicans* strains.

We have changed the text to better reflect the data in Figure 5D. Namely, emphasizing that while the E-cadherin protein levels were relatively unchanged, the organization of the E-cadherin staining (Figure 5E) was affected (Lines: 424-432).

Fig. 1B) legend states: *of a population of unicellular organisms in response to chemical **between organisms. It is unclear what this statement refers to. Please clarify.

We apologize for the ambiguity. The legend has been changed to include the full name of the terms represented by * and ** in Figure 1B.

Also in the figure legend for 1B, it states the transcripts are compared to IECs only – I believe this should be compared to medium only controls?

We apologize for the mistake; the legend has been changed accordingly.

Lines 487-488 state: "The low responsiveness of IECs to *C. albicans* infection may be due to the normally commensal lifestyle within the gastrointestinal tract "*C. albicans* is also commensal

colonizer of the oropharyngeal and urogenital tracts correct? I do not think this is relevant to how these different epithelial cells respond to *C. albicans*.

This sentence has been removed.

Comments by Reviewer 3:

Sprague et al use tandem RNA-sequencing to identify a putative mechanism in both CA and the host IEC resulting in translocation of CA across the mucosal barrier and subsequent Candidemia.

For IEC the authors use a CACO2 derivative C2BBe1 seeded with a laboratory reference strain of CA with samples collected on a reasonable timeline to capture early and late events (0, 0.75, 3, 6, 12, and 24h).

Curiously, the transcriptional response in CA over time was largely independent of the presence of host cells. DEG were identified with DESEQ2, and notable for the exception (to the broad pattern above) of zinc-related genes being more prominent in CA co-cultured with IEC as compared to medium-only. This observation was followed-up by a phenotypic study of zrc1Δ/Δ mutant that did not have deficits in adhesion or hyphae but was notable for reduced translocation. Damage (as measured by LDH) could be rescued in some but not all mutants (e.g., zrc1) by exogenous zinc supplementation. The authors then compare wildtype CA grown without IEC to an ece1Δ/Δ strain with IEC, demonstrating a transcriptional pattern in both consistent with Zinc-starvation (lacking in Wt CA with IEC). The authors conclude based on this work that zinc acquisition from candidalysin-mediated IEC injury is required for subsequent invasion.

In contrast, the transcriptional changes in IEC over time demonstrate a clear difference between CA exposed or not, further a temporal response only in CA exposed. The authors go on to note a pattern of gene expression associated with NFkB activation with damage-capable CA strain co-culture, including a colometric assay of NFkB activation.

The authors ultimately present a model in which candidalysin-mediated damage to the host is required to provide the CA with zinc, and this damage (and loss of barrier integrity) are opposed by NFkB and NFkB activated genes in the IEC. This core model seems supported by their experiments, with the story more compelling and comprehensive from the CA perspective. For the host side, the authors uncover damage-dependent and damage-independent mechanisms. The linkage between NFkB activation and resilience against CA is not fully developed here (and arguably beyond the scope), and the use of immortalized lines rather than (now readily available) organoid-derived IEC are not well noted in the discussion. Nor are these findings well contextualized with the clinical associations with risk for invasive CA infection (steroids, zinc-deficiency in the host). Overall this is a solid story that could use some better contextualization.

We appreciate the positive response and constructive feedback from **Reviewer 3**. We agree that the exact mechanisms behind NFκB activation and resilience against *C. albicans* infection are not described here, but also that it would be out of the scope of the current study. We have included more contextualization for our results and have noted the limitations of our model system using only immortalized cell lines in our Discussion (Lines: 518-525; 589-595). We hope that these changes are satisfactory and increase the relevance of our work.

Figure 3 would ideally include the gene expression data of WT CA without IEC for comparison, as this is a key point in the results and overall model (i.e., that zinc starvation in ece1Δ/Δ is comparable to WT without IEC).

We thank the reviewer for the feedback and agree that having some direct comparison in the main figure would help to emphasize the key point. To avoid adding another 5 graphs for the gene expression data, we have included a heat map into the main figure to directly compare the normalized gene expression in the *ece1*Δ/Δ strain to that of the WT(BWP17) at 24 h for both the medium only and infection conditions (Fig 3F).

The legend for Figure 5C does not reflect the data. I.e., for the WT CA the inhibition of NFkB did not have a significant effect on the loss of TEER. The paired result text more accurately reflects the results.

We have changed the caption for Figure 5C to more accurately reflect the barrier integrity data.

The e-cadherin results (5E) could use a better figure legend and perhaps some effort at quantification (e.g., scoring of e-cadherin organization by a blinded observer) and more than one replicate per condition.

We have changed the caption for Figure 5E to more accurately describe the microscopy data presented.

We have collected images from 3 independent, biological replicates for the E-cadherin staining and scored them using a blinded observer. The median scores (from 1-5) have been included as insets in the representative pictures of Figure 5E.

The extensive use of an immortalized cell line as a surrogate for IEC responses is both reasonable and a major limitation not noted by the authors in the discussion. Particularly given the readily available organoid-derived human IEC (including commercially available via Millipore), and repeatedly demonstrated differences in immortalized cell line IEC as compared to organoid or native host tissue responses, this limitation should at least be clearly acknowledged.

We agree with the point brought up by **Reviewer 3**. We have mentioned this limitation with our study in the Discussion (Lines: 589-595).

The authors note that patient factors are major considerations for eventual risk of invasive CA infection, citing antibiotic exposure. But their own work is poorly contextualized in the discussion. For example, the clinical risks from NFkB inhibition (e.g., steroid use clinically) and poor nutritional states (e.g., zinc deficiency is common in chronic / severe illness) are fitting with the author's experimental findings. Clinically, the risk of zinc deficiency is well-recognized enough to be the basis of recommended care for pediatric patients in the ICU (e.g., https://pubmed.ncbi.nlm.nih.gov/36329878/)

We really appreciate the suggestion from **Reviewer 3**. We have incorporated these points into the Discussion (Lines: 518-525).

Figure 1C and Fig 4C depicts log2 fold change. Either in this figure or another figure the actual FPKM / RPKM values should be shown, not just the fold change.

The mean MRN values used to calculate the log2 fold changes is given in SI Table 1. This has been added to the captions for Fig 1C and Fig 4C.

Figure legends ideally should include the test used (e.g., figure 2, 3, etc do not specify the test used) when a p value is provided.

The information for the statistical tests used was included in the Methods section. We have now moved it to each relevant figure caption to improve the readability of the manuscript. We thank **Reviewer 3** for the constructive feedback.

The article has some awkward phrasing in English and could use a copy-edit to aid in clarity.

The manuscript has been carefully checked again by a native speaker.

Figure 4C could use an x-axis label for clarity.

Figure 4C does have labels for the two columns in the heatmap below the figure. In order to improve clarity and ensure the label is not mistaken for the graph below (Fig 4E) we have moved it above the heatmap on top of the respective columns.

Figure 4E y-axis label ideally would specify NFkB activity rather than the raw reading. The latter is fine for the legend (to explain how activity was estimated) but the former would be clearer for a reader to understand what is shown.

Again, we thank **Reviewer 3** for the constructive comment. We have changed the y-axis label on Figure 4E to improve clarity for the reader.

Ideally, reviewers would be provided access (via a reviewer key) to the raw read data. This can be done while keeping the read data non-public.

We apologize if the Reviewer did not receive access to the raw sequencing data. A link to the raw read data with a reviewer access token was included in the cover letter upon submission, but may not have been made accessible to each reviewer. We should have more effectively provided the information.

The raw sequencing data can be found at the following link:

https://www.ncbi.nlm.nih.gov/geo/query/acc.cgi?acc=GSE237496

With the access token: qjqdocqmtbwzlgp
