# Supplementary material for: Candida albicans translocation through the intestinal epithelial barrier is promoted by fungal zinc acquisition and limited by NFκB-mediated barrier protection
Source: PLoS Pathog. 2024 Mar 1;20(3):e1012031. doi: 10.1371/journal.ppat.1012031 (PMC10907035; doi:10.1371/journal.ppat.1012031)
Supplement: Attachment — Submitted filename: Sprague et al., 2024 - Response letter.pdf [file ppat.1012031.s015.pdf]

Dear Editors,

We would like to thank you and the reviewers for the feedback and positive response to our revised manuscript.

Our manuscript has been reviewed further by two referees, one of which raised two points to be addressed. We have conducted additional experiments to address the first point and included the data in the supplementary material. We have also performed the additional statistical comparison for existing data pointed out by the reviewer.

Furthermore, we have included a new supplementary graph to help with visualization of our data, as suggested by the reviewer. We have also made all relevant text changes to accompany these additions.

Additionally, we have included a new table which includes all data used to generate graphs and figures in the manuscript and re-uploaded our figures after using the PACE digital diagnostic tool.

We have provided a point-by-point response to each comment from the reviewer on the following pages and believe that each point has been sufficiently addressed.

The raw sequencing data has been uploaded to the Gene Expression Omnibus and the data are scheduled to be publicly available December 31st, 2024. Upon acceptance of the manuscript, we will change the release date of the data so that they are publicly available before publication. Additionally, the GEO makes all datasets public as soon as the respective ID is used in a publication, even if it is before the scheduled release date.

We hope that our revised manuscript is satisfactory and can be considered for publication.

We look forward to your response.

Best regards,

Bernhard Hube

Our replies (black) to the reviewers' comments (blue) are below:

***Line numbers refer to the manuscript copy with track changes.**

Part I - Summary

Reviewer #2: The study by Sprague et al has been strengthened by additional experiments and qualifying the interpretation of results. Main new experiments include 1) showing that ECE1-dependent regulation of zinc starvation response genes is mostly rescued by the addition of $ZnSO_4$, 2) the addition of a separate NFkB inhibitor verifying ECE1-independent increase in C. albicans translocation through IECs. 3) However, there are 2 issues that must be addressed.

We thank the Reviewer for the positive feedback to the addition of new qPCR data and in acknowledging the supplementary data with the second NFκB inhibitor.

Part II – Major Issues: Key Experiments Required for Acceptance

Reviewer #2:

1) The authors argue that a more complex culture system would be required to determine whether zinc starvation causes growth defects or translocation defects in the ece1Δ/Δ strain vs the wild type. I do not understand why a complex culture system would be required to explore these phenotypes. For example, is the fungal burden of ece1Δ/Δ increased compared to wild type upon $ZnSO_4$-treated samples shown in SI Figure 3.

In our *in vitro* models for IEC infection, the transcriptional zinc starvation response only occurs after 12 h. Even at these later time points a large portion of the fungal cell population grows atop the host cells as filaments and not invasively, as shown in Figure 2E. This may also contribute to the fact that there is an upregulation of zinc acquisition genes in *C. albicans* during IEC infection, though it is significantly less than in the absence of host cells (SI Fig 1). It is also possible that compensatory activities in zinc mobilization of fungal cells may be sufficient to allow normal growth. It should also be noted that growth defects and fungal burden cannot be quantified by monitoring colony forming units (CFUs) under these conditions as all fungal cells grow as elongated hyphae within hours of the experiments. We have, however, performed further experiments with the *ece1Δ/Δ* mutant and the WT(BWP17) to address this question and included these data in a new supplementary figure along with related data that was already included in the manuscript (SI Fig 4). As readouts, we quantified host-cell damage, fungal translocation, and fungal mass as measured by qPCR (of fungal genomic DNA) to determine whether the increased zinc starvation in the *ece1Δ/Δ* strain results in growth defects that could be rescued by zinc supplementation.

As shown in SI Fig 4C, there was no apparent difference in the overall fungal mass during infection of IECs between the WT(BWP17) and *ece1Δ/Δ* in the absence or presence of added zinc at 24 h. Furthermore, we did not see significant change in the host-cell damage or fungal translocation (SI Fig 4A, B) with and without the addition of exogenous zinc.

We have included the new data (SI Fig 4; Lines: 1153-1163), the new results (Lines: 227-231), and methodology (Lines: 632-644; 653-659; 762-766) have been added to the manuscript.

We would argue that the transcriptional response in the WT(BWP17) and *ece1Δ/Δ* mutant is a more sensitive indicator for zinc starvation in our model as compared to growth. Furthermore, even if these levels of zinc starvation would affect fungal growth our model is likely inappropriate to see reduced growth of invading subpopulations as a consequence of zinc starvation. This has

been added to the Discussion to address these limitations of our models and interpret the new data (Lines: 484-492).

2) This statement is not supported by the data: "The host-cell damage defects of zinc uptake impaired mutants (lacking ZRT101, ZRT2, or PRA1), but not of a zinc detoxification defective mutant (zrc1Δ/Δ) were rescued by addition of 25 μM ZnSO4, a zinc concentration known to promote C. albicans growth while not being toxic (Fig 2D) (28)" The data shows no apparent difference in LDH release levels between untreated and ZnSO4 treatment for zrt101Δ/Δ, zrt2Δ/Δ, or pra1Δ/Δ. The authors instead are interpreting a lack of statistical difference between LDH release in ZnSO4-treated wild type and mutant strains as evidence of rescued LDH release. A direct comparison of ZnSO4-treated vs untreated for these strains would be necessary to make this statement.

We thank the Reviewer for the feedback. We have included comparisons between 'Control' and '25 μM $ZnSO_4$' samples for each strain in our statistical analysis. We have changed the wording and thus modified our statement to more specifically refer to the *ZRT101/ZRT2* double deletion mutant and the *ZRC1* deletion mutant in the respective section of the Results to reflect this (Lines: 204-206) and included the statistical information in the caption for Fig 2D (Lines: 1055-1057).

Part III – Minor Issues: Editorial and Data Presentation Modifications

Reviewer #2: Quantification of SI Fig. 5 would be helpful in visualizing the conclusions of this data.

We have added a graph to SI Fig 6 (previously SI Fig 5) (now SI Fig 6B) of the quantified western blot data and added the information to the respective figure caption (Lines: 1176-1178).
